# Construction of a prognostic model for triple‐negative breast cancer based on immune‐related genes, and associations between the tumor immune microenvironment and immunological therapy

**DOI:** 10.1002/cam4.6176

**Published:** 2023-06-12

**Authors:** Yue Zhu, Lin‐Feng Tao, Jin‐Yan Liu, Yi‐Xuan Wang, Hai Huang, Yan‐Nan Jiang, Wei‐Feng Qian

**Affiliations:** ^1^ Department of Breast and Thyroid Surgery the Affiliated Suzhou Hospital of Nanjing Medical University, Gusu School, Nanjing Medical University Suzhou China; ^2^ Department of Critical Care Medicine the Affiliated Suzhou Hospital of Nanjing Medical University, Gusu School, Nanjing Medical University Suzhou China

**Keywords:** immune‐related genes, prognostic model, triple‐negative breast cancer, tumor immune microenvironment

## Abstract

**Background:**

Triple‐negative breast cancer (TNBC) is the subtype of breast cancer with the worst prognosis, and it is highly heterogeneous. There is growing evidence that the tumor immune microenvironment (TIME) plays a crucial role in tumor development, maintenance, and treatment responses. Notably however, the full effects of the TIME on prognosis, TIME characteristics, and immunotherapy responses in TNBC patients have not been fully elucidated.

**Methods:**

Gene Expression Omnibus and The Cancer Genome Atlas data were used to data analysis. Single‐cell sequencing and tissue microarray analysis were used to investigate gene expression. The concentrations and distributions of immune cell types were determined and analyzed using the CIBERSORT strategy. Tumor immune dysfunction and exclusion score and the IMvigor210 cohort were used to estimate the sensitivity of TNBC patients with different prognostic statuses to immune checkpoint treatment.

**Results:**

Five immune‐related genes associated with TNBC prognosis (IL6ST, NR2F1, CKLF, TCF7L2, and HSPA2) was identified and a prognostic evaluation model was constructed based on those genes. The respective areas under the curve of the prognostic nomogram model at 3 and 5 years were 0.791 and 0.859. The group with a lower nomogram score, with a better prognosis survival status and clinical treatment benefit rate.

**Conclusion:**

A prognostic model for TNBC that was closely related to the immune landscape and therapeutic responses was constructed. This model may help clinicians to make more precise and personalized treatment decisions pertaining to TNBC patients.

## 1 INTRODUCTION

A total of 15%–20% of all breast cancers are triple‐negative breast cancer (TNBC), which lacks expression of the estrogen receptor, progesterone receptor, and human epidermal growth factor receptor 2 (HER‐2).^1^ TNBC has the worst prognosis of all subtypes of breast cancer, so improving the survival rate of TNBC patients has been a hot topic of research. The cornerstone of treatment for TNBC is cytotoxic chemotherapy.^2^ Despite the best efforts of systemic chemotherapy, less than 30% of women with metastatic breast cancer survive for 5 years after their diagnosis.^3^ Ninety percent of TNBCs that remain after chemotherapy have changes in pathways that can be targeted by medicines that are now undergoing clinical research, including poly ADP ribose polymerase (PARP) inhibitors,^4^ phosphoinositide 3‐kinase (PI3K) inhibitors,^5^ and mitogen‐activated protein kinase kinase (MEK) inhibitors.^6^ The recent approval of three targeted therapies for TNBC, such as the two PARP inhibitors olaparib^7^ and talazoparib^8^ for germline BRCA mutation‐associated breast cancer, and atezolizumab in combination with nab‐paclitaxel^9^ for programmed death‐ligand 1 (PD‐L1) + advanced TNBC, indicates that the investigation of TNBC‐targeting biomarkers is currently an area of intense interest.

The tumor immune microenvironment (TIME) plays a key role in the occurrence and development of tumor biology.^10^ With the rise of immunotherapy in recent years, therapeutic strategies such as immune checkpoint inhibitors, active or and passive immunotherapy, and gene therapy have been applied to TNBC.^11^, ^12^, ^13^ In addition, immune molecules in the TIME have great potential as prognostic biomarkers.^14^, ^15^, ^16^ Zhang et al.^17^ reported that as well as having prognostic significance, a set of immune‐related genes in TNBC had important predictive value with respect to immune invasion and the effects of immunotherapy. However, studies to date have not fully elucidated the role of immune genes in TNBC. Identifying potential therapeutic targets for TNBC and evaluating their therapeutic efficacy via changes in the TIME warrants in‐depth study.

In the present study immunological TNBC‐related genes were identified by constructing WGCNA in GSE65194. Hub genes were selected via LASSO and SVM‐RFE machine learning algorithms. A nomogram model was then constructed, and its predictive capacity and clinical accuracy were validated using a dataset derived from The Cancer Genome Atlas (TCGA). Lastly, different TIME statuses among groups with variable nomogram scores were investigated in TNBC patients with diverse prognostic statuses, as were their responses to immunotherapy.

## 2 MATERIALS AND METHODS

### 2.1 Collection of tumor samples

Seven TNBC patients undergoing surgery at the Suzhou Municipal Hospital of Nanjing Medical University, China, between November 2020 and July 2021 provided us with cancer tissues and surrounding tissues used in our prior studies.^18^ All subjects supplied written informed consent, and the study was approved by Suzhou Municipal Hospital's ethics committee. All information pertaining to the participants was kept secure. The samples collected were formalin‐fixed for long‐term storage.

### 2.2 Public gene expression dataset

Data derived from 110 individuals who had been diagnosed with TNBC based on conclusive pathological evidence were obtained from the TCGA database. Gene expression data from 55 patients diagnosed with TNBC, 39 diagnosed with HER‐2, 29 diagnosed with luminal‐A, and 30 diagnosed with luminal‐B were included in a GSE65194 dataset. Ten of the GSE176078 dataset TNBC samples were analyzed via single‐cell RNA sequencing. Data on immune genes were downloaded from the ImmPort data portal, and a total of 2483 genes associated with immunity were found.

### 2.3 Screening of immunological genes related to TNBC

Subtype‐related differentially expressed genes in the GSE65194 dataset were analyzed using “WGCNA” software that is available in the R programming language. When the soft‐thresholding power was equivalent to 6, the correlation coefficient approached 0.9, and scale‐free coexpression networks were constructed. The size of each gene module was limited to ≤50 genes, and cluster analysis was utilized to determine which genes should be grouped together based on similarities in their patterning. To further investigate the gene modules, first a cut‐height criterion of 0.15 was established, then a few of the modules were integrated together. A link between phenotypic eigengenes and module eigengenes served as a foundation for module‐trait interactions. Correlations based on this facilitated the identification of modules that were most closely linked to the TNBC subgroup. The intersection of the IMMPORT gene and the TNBC‐related genes were the immunological genes related to TNBC.

### 2.4 Enrichment analysis

An immune system gene protein–protein interaction network was constructed using “STRING” database. That network was then used to undertake gene ontology (GO) and Kyoto Encyclopedia of Genes and Genomes (KEGG) functional enrichment investigations.

### 2.5 Construction of a predictive prognostic model of TNBC

Univariate Cox analysis of the TCGA dataset indicated a link between levels of immune‐related gene expression and overall survival (OS). *p* < 0.2 was applied as the filtering threshold. Two machine learning techniques, the “LASSO” approach with penalty parameter adjustment performed by 10‐fold cross‐validation and the “SVM‐RFE” algorithm looking for lambda with the least classification error were used to select candidate prognostic immune‐related genes. A predictive signature based on putative immune‐related prognostic key genes was developed using the nomogram model. By calculating the C‐index, the areas under the curves (AUCs) of time‐dependent and combined receiver operating characteristic curves, and the nomogram's calibration curves for 3‐ and 5‐year OS, it was possible to evaluate how accurate the anticipated OS rates were compared with the actual OS rates. Based on the median nomogram score of all TCGA samples, the samples were separated into two groups; one with high nomogram scores and one with low nomogram scores. OS was then compared in groups with low and high points on a Kaplan–Meier survival curve, followed by a log‐rank test. The dataset GSE58812 was used as a validation set to further validate the predictive power of the model. The areas under the curves (AUCs) of time‐dependent and combined receiver operating characteristic curves were used to reflect the predictive ability of the model in validation set.

### 2.6 Immunohistochemistry and semiquantitative analysis

Formalin‐fixed, paraffin‐embedded TNBC tumor and paracancerous specimens from our patients were used for tissue microarray construction. Four‐micrometer‐thick sections were made from the tissue microarray blocks and stained with antibodies specific for chemokine‐like factor (CKLF; cat. no. ab180512; Abcam; 1:100), heat shock protein (HSP) family A member 2 (HSPA2; cat. no. ab108416; Abcam; 1:200), interleukin 6 cytokine family signal transducer (IL6ST; cat. no. 67766‐1‐Ig; Protein Tech; 1:500), nuclear receptor subfamily 2 (NR2F1; cat. no. ab181137; Abcam; 1:100), and transcription factor 7‐like 2 (TCF7L2; cat. no. 13838‐1‐AP; Protein Tech; 1:100). Immunohistochemistry results were evaluated via Image‐Pro Plus 6.0 software. Nonoverlapping fields were randomly picked for each part has been magnified to 40×, and photographs were captured. All photographs were acquired with identical exposure times and white balance settings, and the blank area of the image was used for optical density correction. Brownish‐yellow particles in the cytoplasm were regarded as positive expression of CKLF, NR2F1, IL6ST, HSPA2, and TCF7L2, and expression intensity was assessed by the cumulative value of the integral optical density (IOD) and the area of the target region. The average density (IOD/area) of these fields was used as the analytical index to indicate protein expression.

### 2.7 Tumor immune analysis

“CIBERSORT” is an excellent technique for determining how many immune cells are present in the tumor environment. Samples from TCGA were separated into two groups based on the median nomogram scores, and the infiltration of immune cells in each group was compared.

### 2.8 Assessment of immunotherapy and chemotherapeutic treatment sensitivity

Samples were divided into high and low groups using the median nomogram score. By using the tumor immune dysfunction and exclusion (TIDE) method, the expected responses to the immune checkpoint inhibitors between the different groups were determined by comparing the differences between the two groups after calculating the TIDE scores for each sample. Based on expression of the five key prognostic genes in the model and prognostic data the nomogram score for each sample in the IMvigor210 immune cohort was calculated, and the cohort samples were divided into high‐score and low‐score groups by using the median of all sample scores as the criterion. The prognostic status of different groups was compared by generating Kaplan–Meier survival curves. A scale bar graph was used to show the proportions of complete response (CR)/partial response (PR) and stable disease (SD)/progressive disease (PD) in the different groups of the IMvigor210 cohort, to describe the clinical responses of different groups to immune checkpoint blockade (ICB) immunotherapy. Chemotherapeutic responses in different nomogram score groups were analyzed using “pRRophetic”.

### 2.9 Five key prognostic genes were analyzed at the single‐cell level

The following process was used to analyze the TNBC samples in the single‐cell GSE176078 dataset. Because the data providers have made a clear cell annotation of the original sequencing data,^19^ in the current study the annotation results of different cell clusters were visualized using tSNE and UMAP based on the original annotation. The expression of each immune‐related prognostic gene in different cell clusters was subsequently represented in violin plots. The “Monocle 2R” package was used to establish genealogical distinctions between the various cell subtypes and the possible connections between them at the developmental level. In subsequent pseudotime analysis all T cells were extracted, then cells with mean expression levels greater than 0.1 and dispersion empirical values greater than 1 × dispersion fit were randomly selected. The “DDRTree” method was then used to reduce the dimension of the cells, then the “reduceDimension” function was used to determine the type of cell differentiation state. Lastly, the progression of T‐cell differentiation and the expression levels of the immune‐related prognostic genes in T‐cell developmental trajectories were visualized by using the “plot cell trajectory” function.

### 2.10 Analytical statistics

R (version 4.1.2) was used for all statistical analyses. Differences between groups were assessed via the Wilcoxon test. Unless otherwise specified, *p* < 0.05 was considered statistically significant.

## 3 RESULTS

### 3.1 TNBC‐related immunological genes screened by *WGCNA*

**FIGURE 1:**
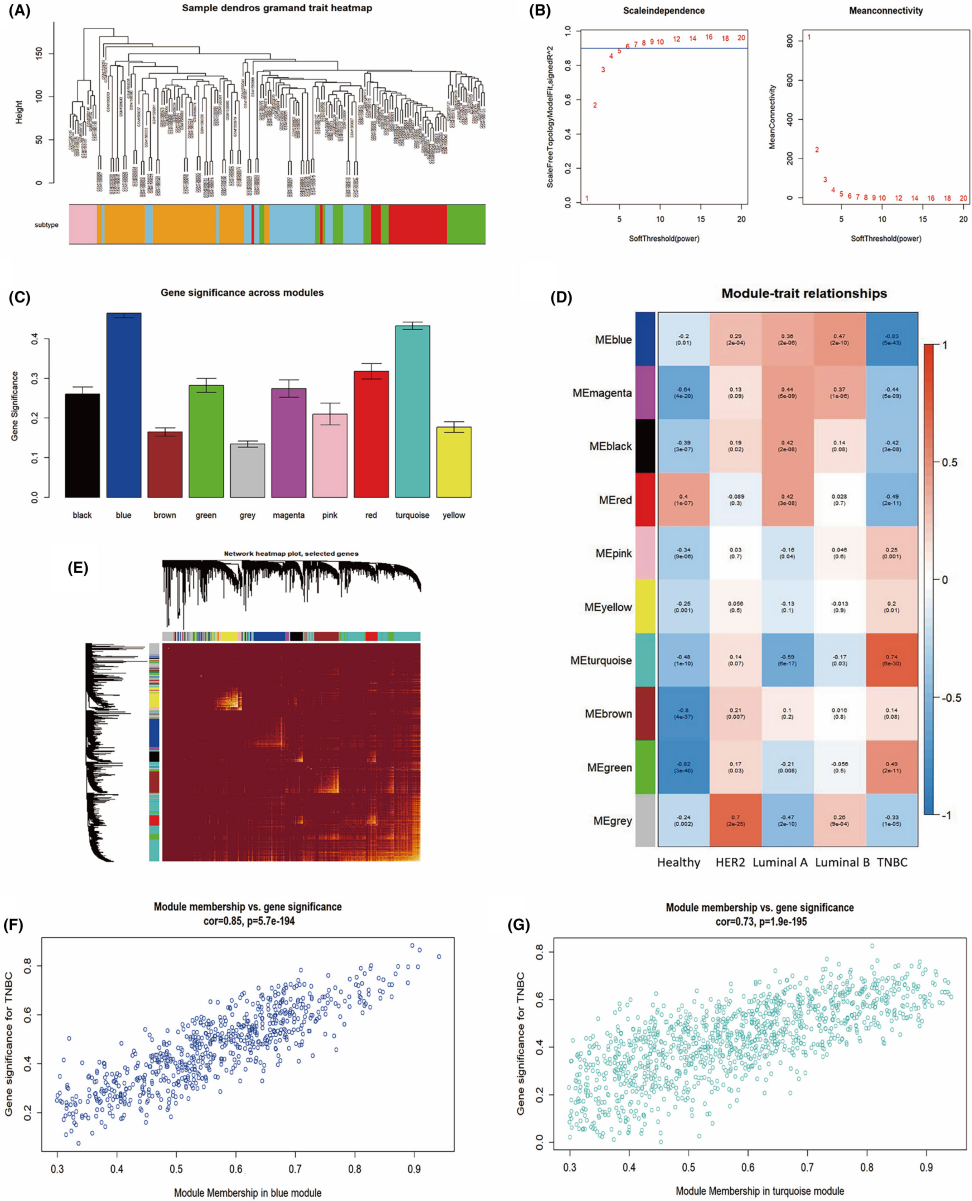
Triple‐negative breast cancer (TNBC)‐related immunological genes screened by *WGCNA*. (A) Samples in GS65194 corresponded to four subtypes of breast cancer. (B) Calculation and selection of optimal soft‐thresholding power. Influence of different powers on scale independence (left) and influence of different powers on mean connectivity (right). (C) Significance levels of 10 coexpression modules associated with TNBC. (D) Relationships between module traits and clinical traits. Each cell contains the corresponding correlation coefficient and *p* value. (E) TOM plot of interactions among coexpression modules. (F) Scatter plots of GS versus MM in the blue modules. (G) Scatter plots of GS versus MM in the turquoise modules.

**FIGURE 2:**
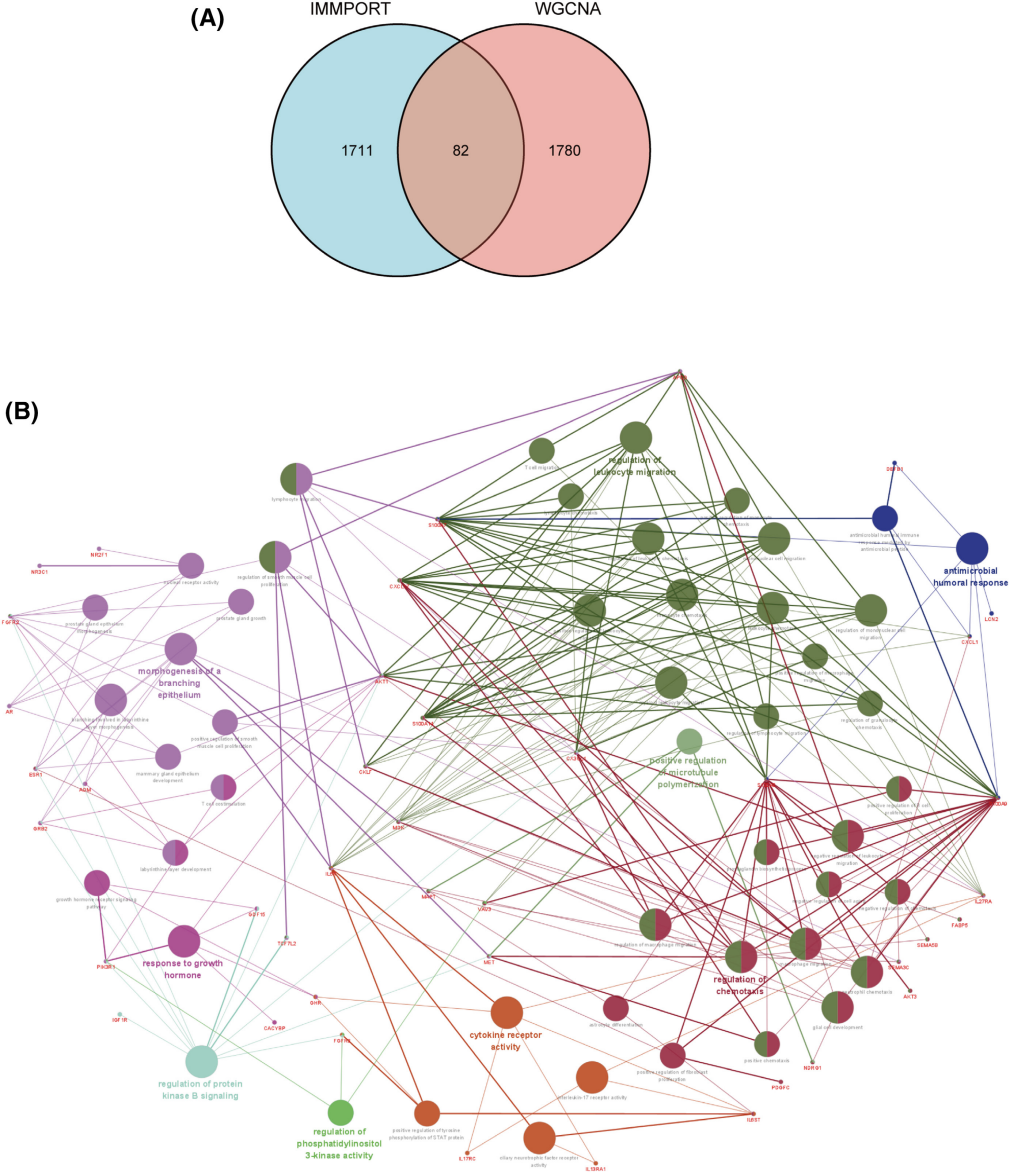
Enrichment analyses of the GO and KEGG databases for immunological TNBC‐related genes. (A) The intersection of the immune‐related genes downloaded from the ImmPort data portal and the TNBC‐related genes, displayed as a Venn diagram. (B) Enrichment analyses of GO and KEGG generated via Cytoscape software.

Gene scale‐free coexpression networks based on *WGCNA* were constructed to investigate the most important TNBC genes. The scale‐free network included all 110 TNBC samples from TCGA. These samples corresponded to four breast cancer subtypes (Figure 1A). The scale‐free topology requirement of *R* ^2^ = 0.9 was used to transform the Pearson correlation matrix between genes into a reinforced adjacency matrix with power = 6 (Figure 1B). Gene coexpression modules were established using average linkage hierarchical clustering and the TOM‐based dissimilarity measure (1‐TOM) for each pair of genes, and a total of 10 modules were identified (Figure 1C). Correlation analysis indicated that the turquoise module included 1171 genes and the blue module included 691 genes, and these were the most significantly representative modules (turquoise correlation = 0.74 and *p* = 6e^−30^; blue: correlation = −0.83 and *p* = 5e^−43^; Figure 1C,D). Clustering dendrograms and modules were used to create a TOM plot for gene networks, and the results revealed that there was no significant association between all modules (Figure 1E). The link between genes and modules is represented by module membership (MM), whereas gene significance (GS) depicts the relationship between genetic and phenotypic features. Scatter plots of the MM and GS relationship in the turquoise and blue modules were generated (turquoise correlation = 0.73 and *p* = 1.9e^−195^; blue correlation = 0.85 and *p* = 5.7e^−194^; Figure 1F,G). Genes highly correlated with traits tended to be important elements significantly associated with the modules. The 2483 immune‐related genes downloaded from the ImmPort data portal were then connected with the 1862 TNBC‐related genes. A total of 82 genes were ultimately selected as TNBC‐related immunological genes for further investigation (Figure 2A).

### 3.2 Enrichment analyses of the GO and KEGG databases

The aforementioned set of 82 genes was investigated using GO and KEGG methods (Figure 2B). The highly enriched genes pertained to growth hormone, regulation of leukocyte migration, antimicrobial humoral responses, cytokine receptor activity, regulation of chemotaxis, and positive regulation of microtubule polymerization. The results were obtained using “ClueGO” and “CluePedia” in Cytoscape software.

### 3.3 Identification of prognostic biomarkers related to TNBC

**FIGURE 3:**
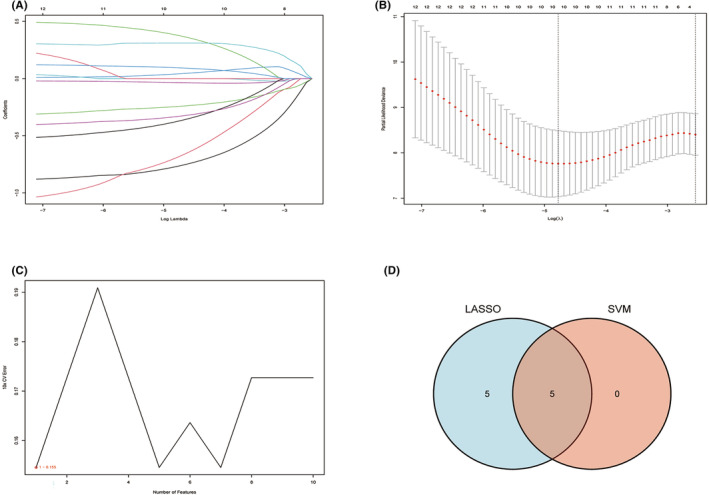
Identification of prognostic biomarkers related to triple‐negative breast cancer. (A, B) Determination of the number of factors by the *LASSO* algorithm. (C) Determination of the number of factors by the *SVM‐RFE* algorithm. (D) Intersection of the *LASSO* and *SVM‐RFE* algorithms, displayed as a Venn diagram.

TCGA transcriptome and clinical data were used to perform univariate Cox regression analysis of the 82 genes identified. The filter criterion used was *p* < 0.2, which yielded 12 genes (Table 1). Twelve genes were identified with the *LASSO* and *SVM‐RFE* algorithms. The *LASSO* algorithm was used to identify 10 nonzero coefficients (Figure 3A,B) by utilizing lambda.min. When the number of features reached 1, 5, and 7, the error of the *SVM‐RFE* algorithm was the lowest (Figure 3C). To simplify the prognostic model, the first five genes of the top features were chosen. Five shared biomarkers for TNBC were defined by overlapping the biomarkers derived from these two algorithms (Figure 3D). These five genes associated with the prognosis of TNBC were considered to be the best, and included IL6ST, NR2F1, CKLF, TCF7L2, and HSPA2.

**TABLE 1:** Results of univariate Cox analysis.

Variable	HR	95% CI	*p* value
TCF7L2	0.399	0.186–0.858	0.019
GPI	2.590	1.170–5.760	0.019
IL6ST	0.494	0.261–0.936	0.031
HSPA2	0.667	0.453–0.983	0.041
SHC2	1.380	0.992–1.920	0.056
RBP1	0.768	0.572–1.030	0.079
VAV3	0.738	0.526–1.040	0.079
MET	0.756	0.551–1.040	0.082
CKLF	0.458	0.188–1.120	0.087
NR3C1	0.565	0.277–1.150	0.115
NR2F1	1.330	0.911–1.940	0.139
S100P	1.150	0.945–1.390	0.166

### 3.4 Construction of an immune‐related prognostic model using the TCGA Dataset

**FIGURE 4:**
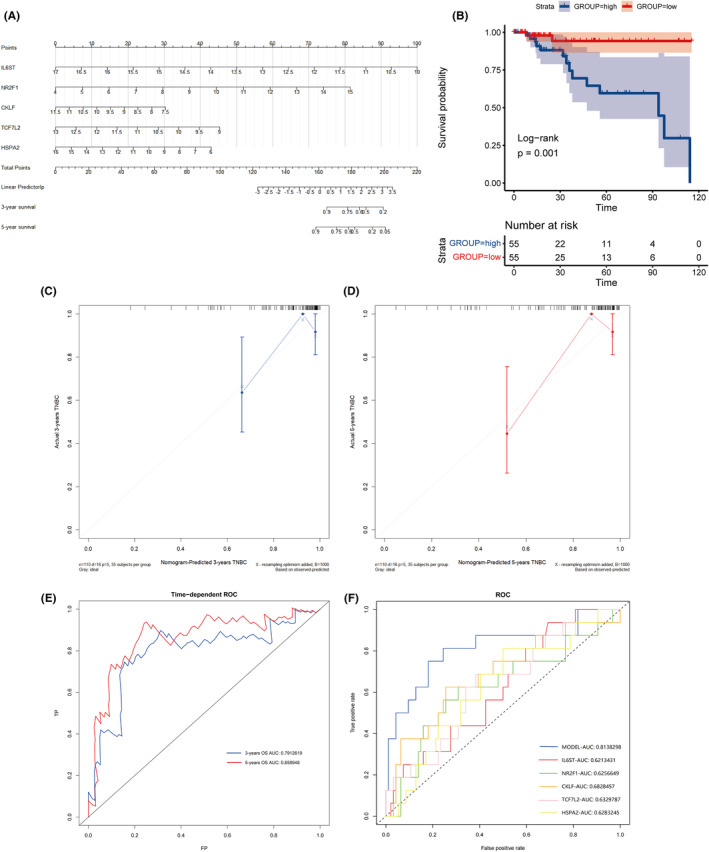
Construction of an immune‐related prognostic model using The Cancer Genome Atlas (TCGA) dataset. (A) Constructed nomogram that depicts the immunological model prognosis in TCGA samples. (B) Kaplan–Meier survival curve of different nomogram score groups. (C, D) Calibration plots of the nomogram at 3 (C) and 5 (D) years. (E) Time‐dependent receiver operating characteristic (ROC) curve of the nomogram. (F) ROC curve of the nomogram and all dependent variables.

**FIGURE 5:**
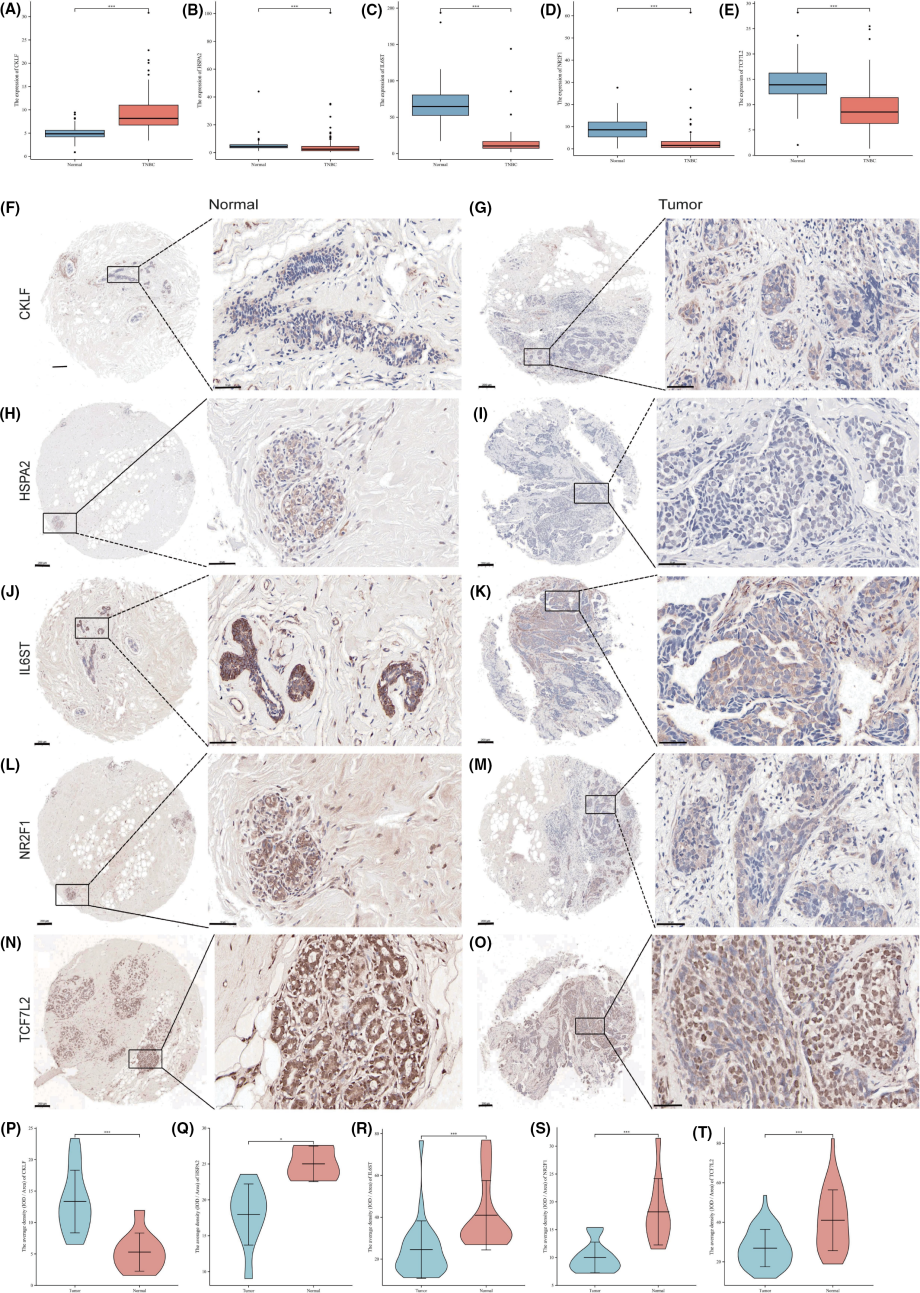
Differences in the expression of the five genes in the model in both paracancerous and tumor tissues from triple‐negative breast cancer (TNBC) samples. (A–E) Differential expression of CKLF (A), HSPA2 (B), IL6ST **(**C), NR2F1 (D), and TCF7L2 (E) in paracancerous and tumor tissues in TNBC using the TCGA database. (F–O) Representative immunohistochemical images depicting the expression of CKLF (F, G), HSPA2 (H, I), IL6ST (J, K), NR2F1 (L, M), and TCF7L2 (N, O) in paracancerous and tumor tissues (4× [left, scale bar = 20 μm] and 40× [right, scale bar = 50 μm] magnification). (P–T) Violin plot showing the average density (IOD/area) of CKLF (P), HSPA2 (Q), IL6ST (R), NR2F1 (S), and TCF7L2 (T) in paracancerous and tumor tissues. *p < 0.05, ***p < 0.001.

*LASSO* and *SVM‐RFE* results from the TCGA dataset were used to create a nomogram that clearly depicts the immunological model prognosis (Figure 4A). Multivariate analysis awarded a score to each variable. All predictive characteristics were taken into account while calculating a total nomogram score. The established OS prediction nomogram had a *C*‐index of 0.769. All 110 TCGA datasets were divided into two groups; a high‐point group (*n* = 55) and a low‐point group (*n* = 55). Survival rates differed significantly between the high‐point and low‐point groups according to the Kaplan–Meier survival curve (*p* = 0.001; Figure 4B). TNBC patient prognosis may be accurately predicted using the nomogram generated in this study, as indicated by the calibration plots of 3 and 5 years (Figure 4C,D). At 3 and 5 years the AUCs of the risk model were 0.791 and 0.859 (Figure 4E). The AUC of the nomogram was 0.814, which was the largest among all dependent variables. The AUCs of IL6ST, NR2F1, CKLF, TCF7L2, and HSPA2 were 0.621, 0.626, 0.683, 0.633, and 0.628, respectively (Figure 4F). According to these data, the nomogram is more accurate for predicting survival in TNBC patients than individual prognostic variables. In the validation set GSE58812, the predictive power of the model was further verified. At 3 and 5 years the AUCs of the risk model were 0.708 and 0.747. The AUC of the nomogram was 0.721, which was also the largest among all dependent variables (Figure S1). Expression levels of the above five genes were compared in normal and TNBC tissues using the TCGA database (Figure 5A–E). Immunohistochemical results indicated differences in expression of the five genes in the model in both normal breast tissue and TNBC tissues (Figure 5F–O). Among them, CKLF (Figure 5P) was upregulated in TNBC tissues, and HSPA2 (Figure 5Q), IL6ST (Figure 5R), NR2F1 (Figure 5S), and TCF7L2 (Figure 5T) were downregulated in TNBC tissues, which is concordant with the results of TCGA database analysis.

### 3.5 Analysis of key prognostic genes at the single‐cell level

**FIGURE 6:**
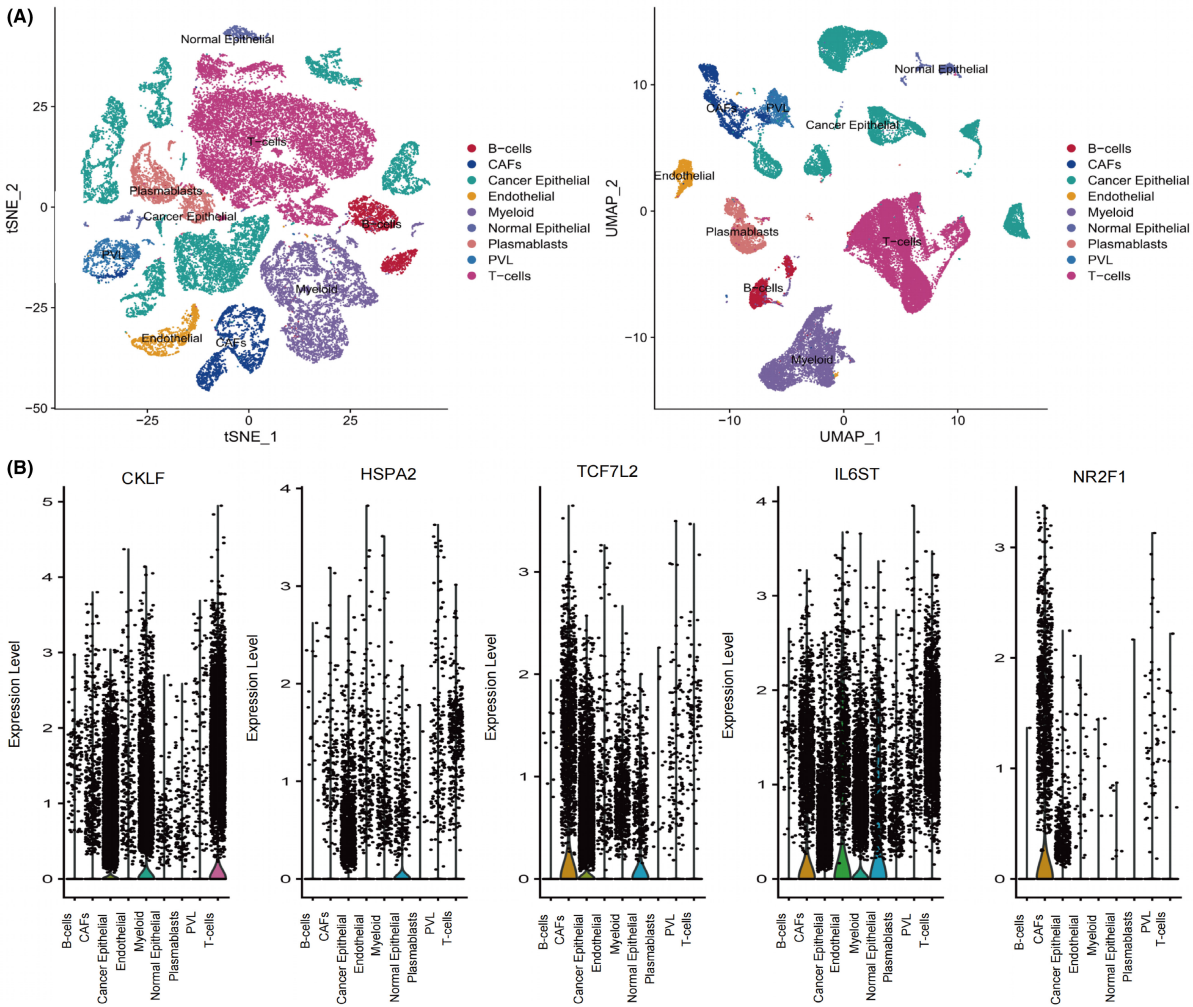
Expression analysis of key genes at the single‐cell level. (A) tSNE and UMAP projections of triple‐negative breast cancer cells in GSE176078. Different cell types are indicated by unique colors. (B) Violin plots depicting the distribution of key genes in cell subpopulations.

**FIGURE 7:**
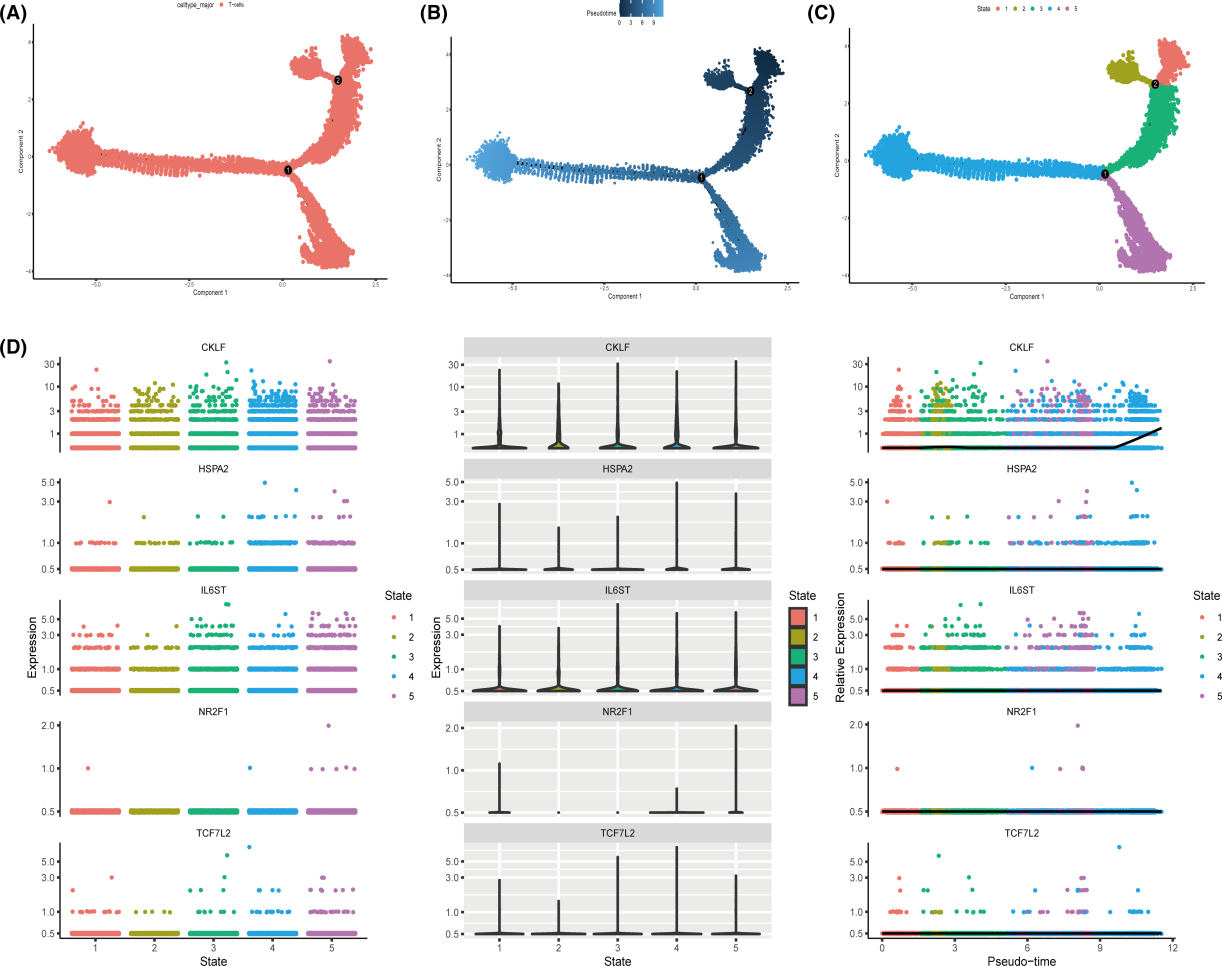
Pseudotime analysis of T cells in triple‐negative breast cancer samples from GSE176078. (A) All cells analyzed were T cells. (B) Timing differences in T‐cell differentiation. Darker blue represents an earlier stage of differentiation, whereas lighter blue indicates a later stage of differentiation. (C) Five stages of T‐cell differentiation. State 1 is the earliest stage of differentiation. (D) Expression levels of key genes at different T cell developmental stages.

Based on cell annotation results from the data provider, cells in TNBC tissues were divided into nine cell subpopulations; B cells, cancer‐associated fibroblasts (CAFs), cancer epithelial cells, endothelial cells, myeloid cells, normal epithelial cells, plasmablasts, perivascular‐like (PVL) cells, and T cells (Figure 6A). CKLF was mainly expressed in CAF, cancer epithelial, myeloid, and T‐cell clusters, HSPA2 was mainly expressed in cancer epithelial, myeloid, and normal epithelial cell clusters, TCF7L2 was mainly expressed in CAF, cancer epithelial, cancer, myeloid, and normal epithelial cell clusters, and IL6ST was mainly expressed in CAF, cancer epithelial, endothelial, myeloid, normal epithelial, and T‐cell clusters (Figure 6B). NR2F1 was mainly expressed in CAF and cancer epithelial cell clusters. A simulation analysis of the cell trajectory differentiation of T cells was then performed (Figure 7A). The darker the blue, the earlier the cell differentiated, thus T cells differentiated from right to left over time, with the darkest blue representing the earliest differentiated cells (Figure 7B). As shown in Figure 7C,T cells exhibited several different states of differentiation each marked with a different color, with red indicating the earliest type of differentiation. An expression‐level plot of CKLF, HSPA2, IL6ST, NR2F1, and TCF7L2 in single‐cell developmental trajectories was then generated (Figure 7D), which indicated that CKLF expression of was upregulated in late T‐cell differentiation. Other genes exhibited no clear changes during the progression of T‐cell differentiation.

### 3.6 Infiltration fraction overview of 20 immune cells across patients

**FIGURE 8:**
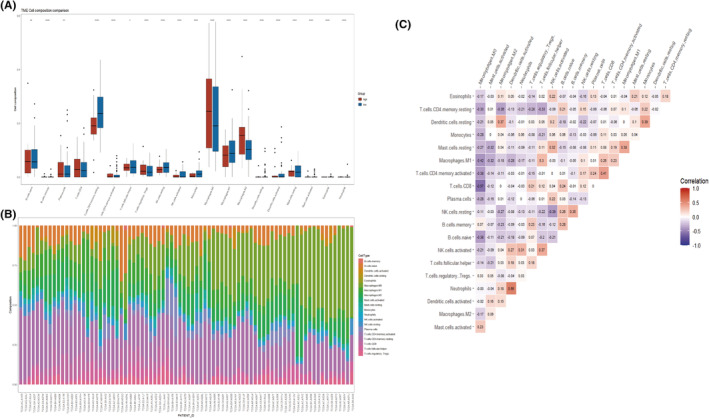
*CIBERSORT* analysis of all patients in The Cancer Genome Atlas dataset. (A) Proportions of TME cells in different nomogram score subgroups. (B) Percentages of immune cells in each triple‐negative breast cancer sample. (C) Correlation matrix of 20 immune cell proportions.

TNBC patients with various prognostic statuses were investigated using the *CIBERSORT* method to categorize immune cell composition and clinical type based on TCGA datasets. Application of a threshold of *p* < 0.05 resulted in the inclusion of 81 samples. Patients in the two categories had unique immune landscapes based on nomogram scores. Compared to the high‐risk group, patients in the low‐risk group (*n* = 63) exhibited significantly larger proportions of resting memory CD4 T cells, follicular helper T cells, resting natural killer cells, monocytes, and M1 macrophages. Regulatory T cells, M0 macrophages, and M2 macrophages were more prevalent in the high‐risk group (*n* = 17; Figure 8A). The color‐coded bars in Figure 8B depict the percentages of immune cells in each TNBC sample. The higher each bar, the more immune cells were present in a given sample. M1 macrophages were positively associated with activated CD4 memory T cells and follicular helper T cells, but were negatively correlated with regulatory T cells, M2 macrophages, and M0 macrophages. Activated CD4 memory T cells were positively associated with CD8 T cells, plasma cells, and M1 macrophages, but were negatively correlated with regulatory T cells, follicular helper T lymphocytes, and M0 macrophages (Figure 8C).

### 3.7 Significance of the TNBC‐related gene signature in immunotherapy

**FIGURE 9:**
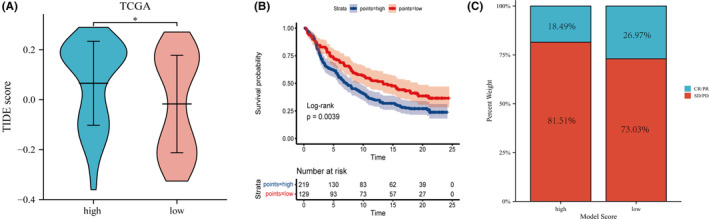
Assessment of the sensitivity and clinical benefit of immunotherapy in the different score subgroups. (A) Tumor immune dysfunction and exclusion analysis of different nomogram score groups in triple‐negative breast cancer samples from The Cancer Genome Atlas. (B) Kaplan–Meier survival curves of different nomogram score groups in the IMvigor210 cohort. (C) Bar graph showing the proportions of complete response/partial response and stable disease/progressive disease in different nomogram score groups in the IMvigor210 cohort.

The clinical effectiveness of immunotherapy in distinct prognostic groups was evaluated using TIDE. Patients who had higher TIDE prediction scores had a lower chance of benefiting from immune checkpoint treatment (ICI). Patients with higher TIDE scores were more likely to benefit from ICIs (Figure 9A). Having a higher TIDE prediction score was related to a lower chance of success. TIDE outcomes perfectly matched prognostic assessment results based on the nomogram model. The immunotherapy cohort IMvigor210 was divided into high‐score and low‐score subgroups. A Kaplan–Meier curve reflected a significant difference in survival prognosis between the two subgroups (Figure 9B), and the prognostic status of the low‐score group was better than that of the high‐score group. This indicates that patients with lower nomogram scores had relatively favorable prognostic outcomes in the population receiving ICB treatment, which is consistent with the aforementioned survival outcomes of derived by analyzing high and low nomogram score subgroups in the TNBC population. In the low‐score group, the CR/PR proportion was 26.97%, and the SD/PD proportion was 73.03%. In the high‐score group, the CR/PR proportion was 18.49%, and the SD/PD proportion was 81.51% (Figure 9C). This indicates that the low‐score group had a better disease remission rate after receiving ICB treatment than the high‐score group.

## 4 DISCUSSION

TNBC is one of the major subtypes of breast cancer with high immune heterogeneity and metastatic potential.^20^ Despite the increasing incidence of TNBC, molecularly targeted drugs and ICB therapy have led to significant improvements in survival in patients with advanced TNBC.^11^ The complexity of TIME in TNBC has led to treatment side effects and drug resistance however, and even relapse in some patients during treatment.^21^ Very recently a single‐cell and spatially resolved transcriptomics analysis deconvoluted large breast cancer cohorts and stratified them into nine ecotypes. In particular, the molecular signatures from PD‐L1/PD‐L2+ macrophage populations, mesenchymal cells, and stromal‐immune niches identified ecotypes with unique cellular compositions and clinical outcomes.^19^ Studies indicate that dynamic changes in neutrophils and macrophages have redefined the “immune subtypes” of TNBC, including neutrophil enrichment subtypes and macrophage enrichment subtypes. These different subtypes respond differently to ICB treatment.^22^ It is therefore necessary to find suitable TNBC therapeutic targets and further evaluate changes in TIME to predict treatment outcomes.

The prognostic immune nomogram model developed in the current study contained five genes; IL6ST, NR2F1, CKLF, TCF7L2, and HSPA2. A member of the interleukin family of cytokines that includes IL6, CNTF, LIF, and OSM, IL6ST is a signal transducer that activates the JAK/STAT and MAPK/PI3K/ERK signaling pathways. The complex signaling mechanisms of IL6ST evidently play critical roles in immunology and a wide range of cancers.^23^ In a previous study higher IL6ST levels were significantly associated with better OS in TNBC patients.^24^ The steroid hormone receptor NR2F1 is an orphan nuclear receptor assumed to be a transcriptional repressor though it may also promote the expression of target genes.^25^, ^26^, ^27^ Breast cancer dormancy gene signatures have recently identified NR2F1 as a gene with increased expression in dormant cells.^28^ NR2F1 is also a strong candidate gene for breast cancer susceptibility, and greater transcript levels are related to resistance to the development of mammary carcinoma.^29^ Neutrophils, monocytes, and lymphocytes are all attracted to CKLF, which is a cytokine and chemoattractant. Ovarian cancer cells have previously been shown to release CKLF, which promotes cancer cell proliferation in an autocrine or paracrine manner, as previously described.^30^ However, few studies have investigated associations between CKLF and cancer. TCF7L2 is one of several transcription factors related to T cell factor/lymphoid enhancer binding factors.^31^ Zheng et al.^32^ reported that the Wnt/−catenin pathway promotes breast cancer stemness via the LUCAT1/miR‐5582‐3p/TCF7L2 axis. That investigation indicated that TCF7L2 is responsible for the transcriptional activation of downstream factors via the TCF7L2/−catenin complex. This implicates TCF7L2 as a crucial component in metabolic processes, cell differentiation, cell proliferation, and cell death.^33^ The molecular chaperone system is mostly comprised of HSPs, which play an important role. A member of the HSP family, heat shock protein A2 (HSPA2), has recently emerged as an important cancer‐related protein with potential biomarker implications.^34^ A recent study investigated novel therapeutic strategies in breast cancer via suppression of HSPA2‐mediated oncogenic signaling by specific small‐molecule inhibitors.^35^ In the present study CKLF was upregulated in TNBC tissues, and NR2F1, IL6ST, HSPA2, and TCF7L2 were downregulated in TNBC tissues, which was concordant with the results of TCGA database analysis and immunohistochemistry results.

Tumor infiltrating lymphocytes (TILs) and immune‐related gene expression signatures provide prognostic and predictive insight into early TNBC.^36^, ^37^ The invasion abundance and spatial distribution of immune cells are also influencing factors.^38^, ^39^, ^40^ In an effort to classify tumor immune microenvironment researchers investigated the numbers of antigen‐producing cells and their spatial distributions, and described four subtypes.^40^ Immune deserts and fully inflamed tumors, respectively, have homogenously low and high numbers of CD8^+^ T cells. Margin‐restricted and stroma‐restricted tumors have no or low numbers of lymphocytes within the tumor, and are characterized by restricted CD8^+^ T cells.^40^ There is a limited infiltrate of immune cells in immune desert tumors, which consists mainly of PD‐L1+ macrophages and exhausted T cells.^41^ Single‐cell RNA sequencing has provided deeper insight into immune cells' complex biology. A single macrophage can coexpress genes associated with M1 and M2, challenging the classical polarization model. The activation and differentiation states of T cells are also evidently continuous.^38^ The role of tissue‐resident memory CD8^+^ T cells in breast cancer immunosurveillance has been investigated.^42^ In that study patients with TNBC who had a CD8^+^ tissue‐resident memory‐related gene signature had better disease‐free survival (DFS) and OS. In this study different groups exhibited different immune microenvironments and were associated with different prognostic outcomes. This suggests that the gene model generated in the study may be useful for assessing TNBC prognoses and immune environments.

Tumor sensitivity to immune checkpoint therapy can be predicted using the TIDE score.^43^ In TCGA datasets in the present study TIDE score was lower in the low‐risk subtype than in the high‐risk subtype. This suggested that patients with lower nomogram scores were more sensitive to ICB treatment. Imvigor210, a clinical cohort that received programmed cell death protein 1 (PD‐1)/PD‐L1 immunotherapy, was used to compare prognoses and clinical efficacy in subgroups with different scores, and the results were consistent with expectations. These findings suggest that a prognostic model based on five specific genes may be useful for evaluating responses to targeted therapy and immunotherapy, which may help promote the development of personalized therapies for TNBC.

In the present study the expression levels of five key genes were analyzed in different cells via single‐cell sequencing. CKLF was significantly enriched in T cells, and visualization of the developmental trajectory of T cells using pseudotime analysis indicated that CKLF was expressed in the late stage of T‐cell differentiation. There is very little research on CKLF, but CMTM4 and CMTM6, which are also members of the human chemokine‐like factor superfamily, reportedly play an important regulatory role in PD‐1/PD‐L1 immunotherapy.^44^ ICB is currently the most advanced cancer immunotherapy. By binding ligands, T cell inhibitory receptors send signals to block T cell activation, preventing autoimmunity. PD‐1 is an immunological checkpoint receptor expressed on antigen‐stimulated T cells. PD‐1/PD‐L1 signaling decreases T‐cell proliferation, cytokine generation, cytotoxicity, and survival. Reactivating T cell‐mediated antitumor immunity is a common goal of cancer therapy, and ICIs such as anti‐PD‐1 and anti‐PD‐L1 monoclonal antibodies are frequently used to achieve this goal.^45^ Because most patients with cancers cannot benefit from PD‐1/PD‐L1 immunotherapy due to congenital or acquired resistance, there is a need to identify target genes that can be mutually regulated with PD‐1/PD‐L1 to improve the benefit rate of clinical immunotherapy, and we suspect that CKLF may play the above‐described regulatory role, but this needs to be confirmed by further experiments.

While the findings of the current study are valuable, the study had some limitations. A public database was used to generate constructs and validate the results. Therefore, prospective studies are essential to evaluate the clinical application of the model in patients with TNBC. Comprehensive functional experiments are essential to elucidate the specific mechanisms associated with the genes involved.

## 5 CONCLUSION

A nomogram model for predicting survival and immunological responses was constructed based on the five immune‐related genes identified in this investigation. Relationships between TNBC prognosis and dynamic changes in the tumor microenvironment were evaluated.

## AUTHOR CONTRIBUTIONS

**Yue Zhu:** Formal analysis (equal); methodology (equal); software (equal); writing – original draft (lead). **Lin‐Feng Tao:** Formal analysis (equal); methodology (equal); software (equal). **Jinyan Liu:** Methodology (equal); validation (equal); writing – review and editing (equal). **Yi‐Xuan Wang:** Data curation (equal); methodology (equal); resources (equal). **Hai Huang:** Funding acquisition (lead); investigation (equal); methodology (equal); writing – review and editing (equal). **Yannan Jiang:** Project administration (equal); supervision (equal); writing – review and editing (equal). **Wei‐Feng Qian:** Project administration (lead); resources (equal); supervision (lead).

## FUNDING INFORMATION

The Suzhou Health and Family Planning Commission (SKJYD2021104).

## CONFLICT OF INTEREST STATEMENT

The authors have disclosed no conflicts of interest.

## ETHICS STATEMENT

The authors are accountable for all aspects of the work in ensuring that questions related to the accuracy or integrity of any part of the work are appropriately investigated and resolved. Signed informed consent regarding this study was provided by all participants, and ethical approval for the study was received from the Suzhou Municipal Hospital. The application of the TCGA and GEO databases was approved by the review boards of the Massachusetts Institute of Technology and Beth Israel Deaconess Medical Center. The study was conducted in accordance with the Declaration of Helsinki (as revised in 2013).

## Supporting information


Figure S1.

## Data Availability

All data generated or analysed during this study are included in this published article [and its supplementary information files].
