# Supplementary figures and images for: Construction of a prognostic model for triple‐negative breast cancer based on immune‐related genes, and associations between the tumor immune microenvironment and immunological therapy
Source: Cancer Med. 2023 Jun 12;12(14):15704–19. doi: 10.1002/cam4.6176 (PMC10417082; doi:10.1002/cam4.6176)

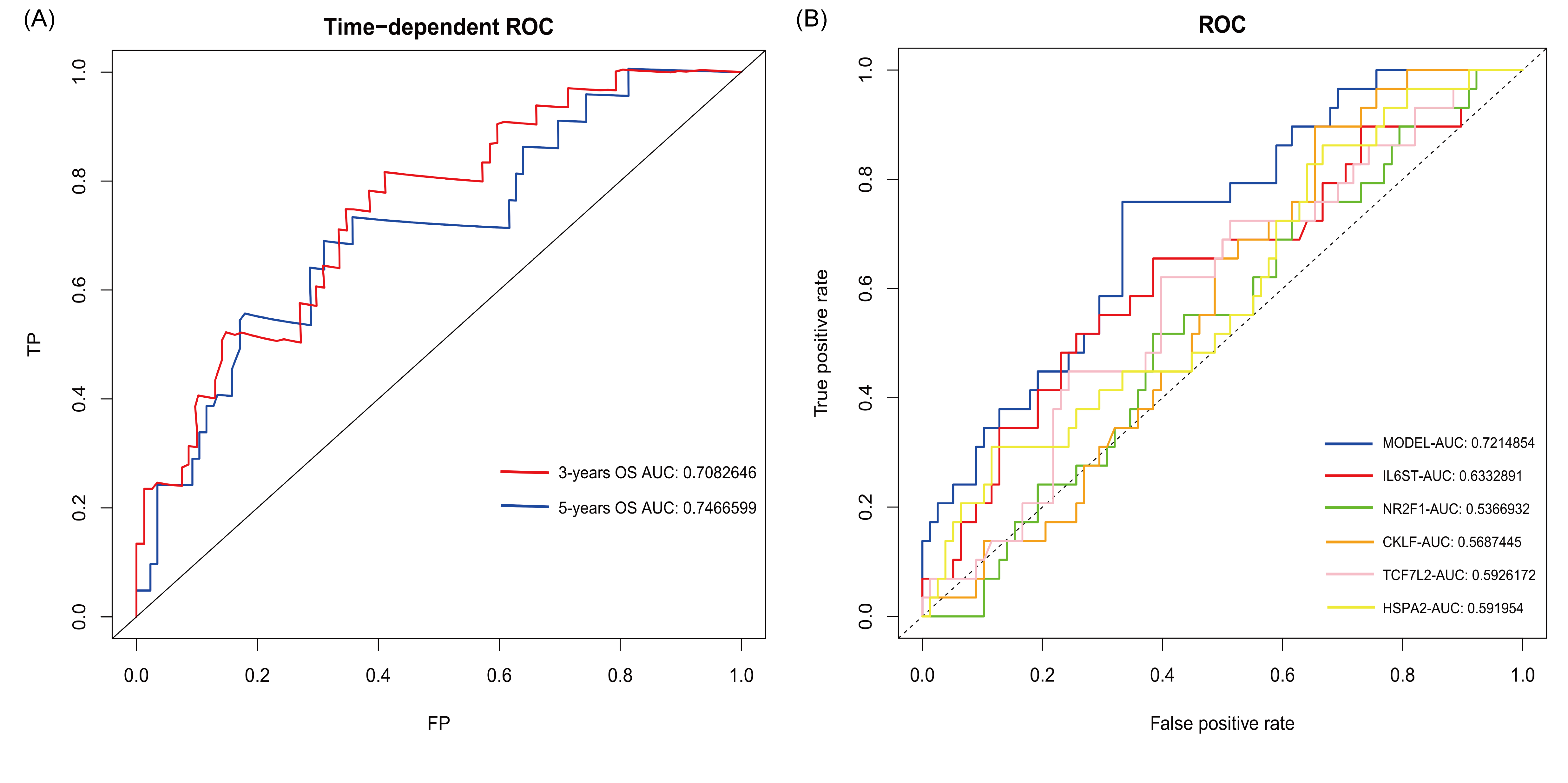

Supplement: Supplementary file 1 — Figure S1. [file CAM4-12-15704-s001.tif]
